# Development of a Machine Learning-Based Predictive Model and Clinically Oriented Web Application for 30-Day Mortality Following Cardiac Surgery

**DOI:** 10.3390/s26051656

**Published:** 2026-03-05

**Authors:** Telmo Miguel-Medina, Susel Góngora Alonso, Isabel de la Torre Díez, Miriam Blanco Sáez, Hector Lazaro Arrechea Elissalt, Atenea Ruigómez Noriega, María Lourdes del Río Solá

**Affiliations:** 1 eHealth and Telemedicine Group (GTe), University of Valladolid, 47011 Valladolid, Spain; 2 Angiology and Vascular Surgery Department, University Hospital of Valladolid, 47003 Valladolid, Spain; 3 Universidad Europea del Atlántico, 39011 Santander, Spain; 4 Universidad Internacional Iberoamericana, Campeche 24650, Mexico; 5 Universidad Internacional Iberoamericana, Arecibo 00613, Puerto Rico; 6 Universidade Internacional do Cuanza, Cuito EN250, Angola; 7 Fundación Universitaria Internacional de Colombia, Bogota 110911, Colombia; 8 Universidad de La Romana, La Romana 22000, Dominican Republic; 9 Faculty of Health Science, University of Valladolid, 42005 Valladolid, Spain; 10 Faculty of Health Science, European University Miguel de Cervantes, 47012 Valladolid, Spain

**Keywords:** mortality prediction, cardiac surgery, machine learning, XGBoost, clinical decision support, explainability, web application

## Abstract

This study aimed to develop and validate a machine learning-based model for predicting 30-day mortality in cardiac surgery patients and to implement a functional, clinician-oriented web application that enables the real-time use of the model. A retrospective cohort of 325 cardiac surgery patients was analysed using supervised machine learning. After preprocessing and clinical feature selection, several models were trained and evaluated through cross-validation. XGBoost achieved the best results, with an AUC-ROC of 0.968, recall of 0.800, and Brier score of 0.058. To facilitate clinical usability, a web-based application was developed using StreamLit, enabling clinicians to input patient data and predict mortality in real time. The application includes SHAP-based explainability for each prediction, thereby ensuring model transparency. Preliminary feedback from clinicians indicated that the tool was intuitive and informative and showed potential for preoperative risk assessment. The integration of a robust ML (machine learning) model with a functional clinical application offers a practical tool for supporting decision-making in cardiac surgery. This combined approach enhances both accuracy and accessibility, which are key to real-world impacts. Future work will involve multicentre validation and user-centred refinement.

## 1. Introduction

The early prediction of mortality in patients with cardiovascular diseases is an area of growing interest in both clinical medicine and health research. Cardiovascular diseases remain the leading cause of death worldwide, according to the World Health Organization [1], and represent a substantial burden on healthcare systems and affected families. In this context, accurately estimating the risk of death within a short time frame—such as the first 30 days after hospital admission—is essential to guide clinical decisions, optimise resource allocation, and improve health outcomes. Over the past decades, various predictive models have been developed using classical statistical techniques such as logistic regression, particularly in cohorts of patients with heart failure, acute myocardial infarction, or coronary artery disease. These models, including TIMI and GRACE [2,3], have been widely implemented in clinical practice.

Recent machine learning (ML) approaches for 30-day mortality post-cardiac surgery have shown promise over traditional scores by capturing non-linear interactions. For instance, XGBoost models on multicentre cohorts achieved an AUC-ROC of up to 0.92 [4], while random forests outperformed EuroSCORE II on single-centre data with sparse events [5]. Unlike population-based scores like GRACE/TIMI, which provide averaged risk strata, ML enables patient-specific predictions incorporating high-dimensional data, enhancing personalisation for individualised interventions such as preoperative optimisation.

However, they exhibit limitations in generalisability, as they are often trained on specific populations, and their performance may decline in settings with different characteristics. Furthermore, they typically assume linear relationships between variables, which may not fully capture the complexity of underlying pathophysiological processes. With advances in data science and machine learning (ML), it has become possible to develop more flexible and powerful models capable of capturing non-linear relationships and complex patterns in clinical data. Models such as decision trees, random forests [5], XGBoost [4], and neural networks [6] have demonstrated improved predictive accuracy compared to traditional approaches. Recent studies have shown that integrating electronic health records with ML algorithms can enhance short-term mortality prediction in patients with cardiovascular conditions by incorporating a wide range of clinical, demographic, and laboratory variables. Despite these advances, the implementation of ML models in clinical practice remains limited. Key barriers include the lack of transparency—particularly in “black-box” models—and concerns regarding generalisability beyond the original training environments. Therefore, it is essential to develop and evaluate predictive models that not only achieve high performance but are also interpretable and rigorously assessed for bias, sample representativeness, and external validity. In this work, we address this need by developing a 30-day mortality prediction model for patients undergoing cardiac surgery, using real-world clinical data and state-of-the-art data science techniques. Emphasis is placed on cross-validation, model interpretability, and bias assessment. Additionally, we deploy the model through a clinician-friendly web application to facilitate its integration into routine clinical workflows.

### 1.1. State of the Art

#### 1.1.1. PRISMA-Based Literature Review

A structured literature review was conducted to identify recent models that predict 30-day mortality after cardiac surgery using machine learning. The review followed the PRISMA guidelines and the sources included Semantic Scholar, Sci-Space, and Paper Digest. Inclusion criteria:

- Publications from 2020 onward;
- Dataset size ≥ 300 patients;
- At least one ML model evaluated;
- Multiple performance metrics supported.

A total of 18 studies were selected after screening [4,5,7,8,9,10,11,12,13,14,15,16,17,18,19,20,21], from which model types, performance scores, and dataset characteristics were extracted to compare with our model.

The systematic review confirms that machine learning models are a promising approach for predicting 30-day mortality (30DM) in patients with cardiac conditions. Algorithms such as XGBoost, random forest, and logistic regression consistently showed strong discriminative and calibration performance, particularly when evaluated using robust metrics like AUC-ROC, sensitivity, specificity, and Brier score. Common predictive variables across studies included age, heart rate, creatinine levels, vasopressor use, and presence of anaemia, emphasizing the need for structured and comprehensive electronic health records. However, a major limitation among most reviewed studies was the lack of external validation, which restricts the generalisability of their findings. Overall, while ML models show potential, further prospective and externally validated research is necessary to ensure their clinical utility and ethical deployment in diverse real-world settings.

This review was designed to provide a structured overview of existing 30-day mortality models rather than a formal meta-analysis. Given the heterogeneity in populations, model types and reported metrics, we did not pool effect sizes; instead, we extracted model types, performance metrics and dataset characteristics to qualitatively position our proposed model within the existing literature.

#### 1.1.2. Handling Class Imbalance and Rare Events

Most of the 30-day mortality studies reviewed above face severe outcome imbalance, yet only a subset explicitly report the use of resampling or class-weighting strategies. Beyond specific cardiac cohorts, there is a growing methodological literature on resampling for rare events in clinical risk models. Yang et al. [22] proposed diversity-aware oversampling to improve mortality prediction in the Surgical Outcome Risk Tool (SORT), showing that combining multiple resampling methods while preserving sample diversity enhances detection of rare deaths and external generalisability. At the same time, several studies have warned about potential harms of naïve imbalance corrections. Van der Ploeg et al. [23] showed that common imbalance corrections for logistic regression did not improve AUROC but led to systematic overestimation of risk and miscalibration of predicted probabilities. Similarly, Welvaars et al. [24] found that resampling could increase apparent discrimination for some machine learning algorithms, yet often produced poorly calibrated models that over-predicted events.

A recent scoping review on resampling methods for class imbalance in clinical prediction models [25] highlighted that data-level techniques (random over-/undersampling, SMOTE and variants) and algorithm-level approaches (cost-sensitive learning) have heterogeneous and context-dependent effects and emphasised the need to report both discrimination and calibration. More broadly, outcome class imbalance has been recognised as an underappreciated threat to fair and reliable risk prediction, as it can inflate overall accuracy while degrading positive predictive value and minority-class performance if inappropriate metrics or correction strategies are used [26].

In our study, given the single-centre cohort (n=325, 8% events), we therefore opted not to use synthetic oversampling (e.g., SMOTE) and instead relied on class-weighted algorithms and threshold optimisation on the original data distribution. This choice is now explicitly discussed in Section 2 and Section 6.

## 2. Materials and Methods

This is a single-centre retrospective cohort study aimed at developing and internally validating a supervised machine learning model for predicting 30-day mortality after cardiac surgery. The methodological pipeline comprised five stages: data acquisition and preprocessing, feature selection, model development, performance evaluation and model selection, and deployment in a web application. Each stage is detailed in the subsections below.

**Figure 1:**
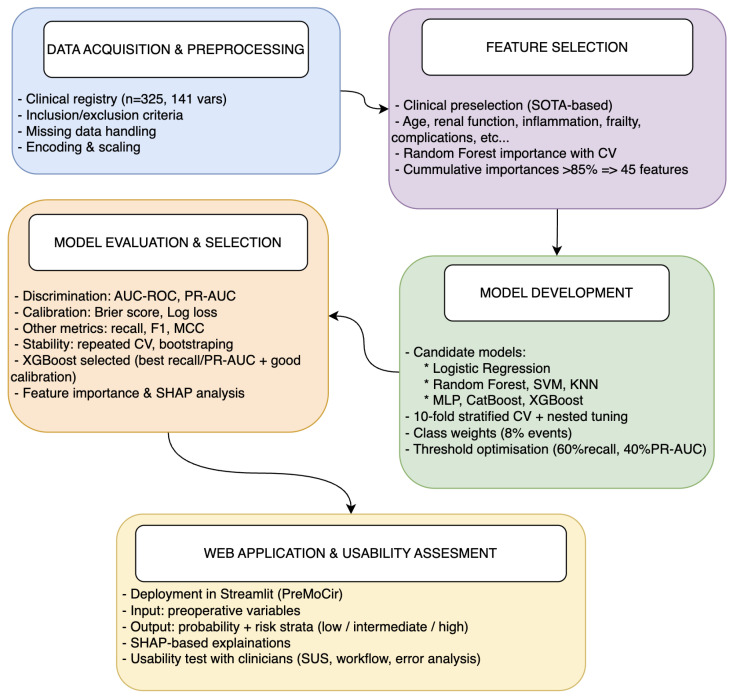
Overall methodological workflow. The pipeline comprises five main stages: (1) data acquisition and preprocessing; (2) feature selection combining clinical judgement and random forest importance; (3) model development with stratified cross-validation and hyperparameter tuning; (4) model evaluation and selection based on discrimination, calibration and recall/PR-AUC; and (5) deployment of the final XGBoost model in the PreMoCir web application.

Figure 1 summarises the methodological workflow followed in this study, from data acquisition to web application deployment, and provides a visual overview of the main steps detailed in Section 2.1, Section 2.2, Section 2.3 and Section 2.4.

### 2.1. Data Analysis

Data were collected prospectively at the University Hospital of Valladolid (HCUV) as part of a clinical registry of cardiac surgery patients.

The dataset includes 325 records and 141 candidate variables. Given the small sample size for machine learning, stratified cross-validation was essential to avoid overfitting. Clinical variables included demographics, lab values, comorbidities, preoperative assessments, and intraoperative data.

After clinical screening and statistical preprocessing, 26 clinically meaningful predictors were retained for model development. These 26 variables (10 continuous and 16 categorical) constituted the input feature set for all machine learning models; after one-hot and ordinal encoding, they expanded to 45 columns in the design matrix.

Missing data were handled using median imputation for numerical variables (overall missingness < 5%, maximum per variable 3.2% for creatinine) and mode for categorical variables; features with >20% missingness were removed (n=2). Categorical features were one-hot-encoded and numerical variables standardised using StandardScaler (mean = 0, std = 1).

Feature selection followed two steps. First, a cardiology expert pre-selected clinically plausible variables based on prior evidence from the 30-day mortality literature (age, renal function, inflammatory markers, frailty indices, and major postoperative complications), informed by our systematic review. Second, within each outer cross-validation fold we fitted a random forest on the pre-filtered set and ranked variables by mean decrease in impurity; predictors were added in descending importance until ≥85% of cumulative importance was reached, yielding 45 features used for all downstream models.

Despite the limited sample size (n = 325), generalisability potential is supported by rigorous internal validation techniques, including stratified 10-fold cross-validation with nested hyperparameter tuning, repeated CV (10 repeats), and bootstrapping (n = 100). These methods provide robust performance estimates and mitigate overfitting, as evidenced by minimal training–validation gaps (AUC-ROC: 0.975 vs. 0.968) and stable feature importance (top-5 correlation r = 0.85 across folds). While external validation remains essential, such approaches align with successful single-centre ML studies in cardiac surgery (e.g., Kanani et al. [5], AUC = 0.92 on similar cohorts).

### 2.2. ML Model Development

Let $X\in R^{n\times p}$ denote the matrix of predictors after preprocessing, with n=325 patients and p=45 encoded features derived from 26 clinically selected variables, and let $y\in {\left\{0,1\right\}}^{n}$ be the binary outcome indicating 30-day mortality ($y_{i}=1$ if patient *i* died within 30 days and $y_{i}=0$ otherwise).

#### 2.2.1. Feature Set and Preprocessing Pipeline

Clinical variables were selected in two stages. First, a cardiology specialist defined a core set of clinically plausible predictors based on prior evidence from the 30-day mortality literature and local expertise. This screening yielded 26 variables: ten continuous (age, body mass index, preoperative haematocrit, leukocyte count, preoperative creatinine, total proteins, C-reactive protein, handgrip strength, 5-metre walk test, and preoperative length of hospital stay) and sixteen categorical (sex, diabetes, smoking, hypertension, dyslipidaemia, obesity, COPD, preoperative renal insufficiency, any postoperative complication, MACE, two binary frailty flags based on grip strength and gait speed, and four frailty/functional scores: Barthel, Katz, FRAIL and Edmonton). Binary predictors were encoded as 0/1 indicators, and frailty/functional scores were treated as ordered categorical variables.

Second, a unified preprocessing pipeline was defined to handle missing values, scale continuous variables and encode categorical predictors. Let $x_{i}^{\left(\mathrm{num}\right)}\in R^{10}$ denote the continuous part of the feature vector for patient *i* and $x_{i}^{\left(\mathrm{cat}\right)}$ the categorical components. Missing values in each numerical feature *j* were imputed by the most frequent observed value in that feature, i.e.,

$$x_{ij}^{\left(\mathrm{num},\;\mathrm{imp}\right)}=\left\{\begin{array}{cc} x_{ij}^{\left(\mathrm{num}\right)}, & \mathrm{if}\;x_{ij}^{\left(\mathrm{num}\right)}\;\mathrm{is}\;\mathrm{observed},\\ \mathrm{mode}\{x_{1j}^{\left(\mathrm{num}\right)},\ldots ,x_{nj}^{\left(\mathrm{num}\right)}\}, & \mathrm{if}\;x_{ij}^{\left(\mathrm{num}\right)}\;\mathrm{is}\;\mathrm{missing}, \end{array}\right.$$

and then standardised to zero mean and unit variance using the mean $\mu_{j}$ and standard deviation $\sigma_{j}$ estimated on the training data:

$$z_{ij}=\frac{x_{ij}^{\left(\mathrm{num},\;\mathrm{imp}\right)}-\mu_{j}}{\sigma_{j}},\;j=1,\ldots ,10.$$

Categorical binary variables were imputed with their most frequent category and kept as 0/1 indicators. Ordered scores (Barthel, Katz, FRAIL, and Edmonton) were imputed with the most frequent value in each score and mapped to integer levels preserving their natural order, for example

$$\mathrm{Barthel}\in \left\{0,1,\ldots ,100\right\}\mapsto \left\{0,1,\ldots ,L_{\mathrm{Barthel}}\right\},$$

where larger values indicate better functional status. After this preprocessing, each patient was represented by a vector ${\tilde{x}}_{i}\in R^{p}$ combining standardised continuous variables, binary indicators and encoded ordinal scores. The 26 clinically selected variables expanded to p=45 encoded features in the design matrix used for model training.

All preprocessing steps (imputation, standardisation and encoding) were defined as part of a single modelling pipeline and estimated using only the training data within each cross-validation fold to avoid information leakage into the validation sets. The implementation was carried out in Python 3.9 using standard machine learning libraries, but the procedures follow the general transformations described above.

#### 2.2.2. Candidate Algorithms and Class Imbalance Handling

On top of the shared preprocessing pipeline, we evaluated four supervised learning algorithms for predicting 30-day mortality: logistic regression (LR), random forest (RF), support vector machine (SVM), and extreme gradient boosting (XGBoost). These methods were selected because they are widely used in clinical prediction studies and offer complementary bias–variance profiles: LR as a linear, interpretable baseline; RF and XGBoost as tree-based ensemble methods capable of modelling non-linear interactions; and SVM as a margin-based classifier suitable for high-dimensional feature spaces. Additional models (*k*-nearest neighbours, multilayer perceptron and CatBoost) were explored in preliminary analyses but were not retained as primary candidates given their inferior performance or less favourable calibration on this dataset.

Given the strong class imbalance in the outcome (8% deaths vs. 92% survivors), we did not employ synthetic resampling techniques such as SMOTE in order to preserve the original joint distribution of predictors and avoid generating artificial patient profiles. Instead, we addressed imbalance at the algorithmic level by using class-weighted loss functions for all main classifiers. If $\pi_{1}$ denotes the prevalence of deaths and $\pi_{0}=1-\pi_{1}$ the prevalence of survivors, class weights were defined as

$$w_{1}=\frac{1}{2\pi_{1}},\;w_{0}=\frac{1}{2\pi_{0}},$$

so that, in expectation, each class contributed equally to the empirical risk. These weights were incorporated into the optimisation of LR, RF, SVM and XGBoost, effectively penalising misclassified deaths more heavily than misclassified survivors and improving sensitivity to the minority class without distorting the data.

All algorithms were implemented in Python 3.9 using standard machine learning libraries, but the modelling strategies described above are independent of the specific software framework used.

#### 2.2.3. Cross-Validation, Hyperparameter Tuning and Threshold Optimisation

Model performance was assessed using stratified *K*-fold cross-validation with K=10. The dataset was partitioned into ten folds of approximately equal size while preserving the outcome prevalence in each fold. For each outer fold *k* (k=1,…,10), the full pipeline (preprocessing + classifier) was trained on the remaining nine folds and evaluated on fold *k*, yielding out-of-fold predicted probabilities ${\hat{p}}_{i}^{\left(k\right)}$ for all patients in the validation fold. This procedure produced a set of out-of-fold predictions ${\left\{{\hat{p}}_{i}\right\}}_{i=1}^{n}$ covering the entire cohort, which were used to compute cross-validated performance estimates.

Hyperparameters were selected via nested 10-fold cross-validation within each outer training set using exhaustive grid search. To reflect the clinical priority of detecting as many deaths as possible while maintaining a reasonable precision, the inner-loop optimisation targeted a composite objective:

J=0.6·Recall+0.4·PR-AUC,

where recall and PR-AUC are defined in Section 2.2.4. The weights 0.6 for recall and 0.4 for PR-AUC were chosen a priori in agreement with clinical collaborators to slightly favour sensitivity over overall precision–recall balance. For each outer fold, the hyperparameter combination that maximised *J* in the inner loop was selected, and the corresponding model was retrained on the full outer training data and applied to the outer validation fold.

Continuous predicted probabilities were converted into binary classifications by thresholding at a value t∈[0,1]. Rather than fixing t=0.5, we tuned probability thresholds post hoc within each outer fold by sweeping *t* and selecting the value that maximised the same composite objective *J* on the corresponding training predictions. The final reported classification metrics were computed at the mean optimal threshold across folds. This procedure ensured that the decision rule was aligned with the clinical preference to minimise false negatives while controlling the proportion of false positives.

#### 2.2.4. Performance Metrics

For each model and each outer fold, we derived confusion matrix components (true positives TP, false positives FP, true negatives TN, and false negatives FN) and computed standard binary classification metrics:

$$\mathrm{Recall}=\frac{TP}{TP+FN},\;\mathrm{Precision}=\frac{TP}{TP+FP},\;F_{1}=2\cdot \frac{\mathrm{Precision}\cdot \mathrm{Recall}}{\mathrm{Precision}+\mathrm{Recall}}.$$

Discrimination was assessed through the area under the receiver operating characteristic curve (AUC-ROC) and the area under the precision–recall curve (PR-AUC). Calibration was evaluated using the Brier score,

$$\mathrm{Brier}=\frac{1}{n}\sum_{i=1}^{n}{\left({\hat{p}}_{i}-y_{i}\right)}^{2},$$

and the log loss,

$$\mathrm{Log}\;\mathrm{loss}=-\frac{1}{n}\sum_{i=1}^{n}\left[y_{i}\log\left({\hat{p}}_{i}\right)+\left(1-y_{i}\right)\log\left(1-{\hat{p}}_{i}\right)\right].$$

We also report the Matthews correlation coefficient and Hamming loss as global measures of classification quality. All metrics are reported as the mean and standard deviation across the 10 outer folds, using out-of-fold predictions.

#### 2.2.5. Relation Between Features, Model and Evaluation Metrics

In our framework, features used to *predict* 30-day mortality correspond exclusively to the clinical variables described above (demographics, laboratory values, comorbidities, frailty scores and complications). Performance metrics such as AUC-ROC, PR-AUC, Brier score, log loss, recall, precision or F1-score are not used as inputs to the model; they are computed *after* training and represent purely evaluative quantities. The machine learning model $f(x_{i})$ estimates an individual probability of death from the clinical feature vector, whereas the metrics summarise how well that function separates deaths from survivors across the cohort.

### 2.3. Application Development

Once the machine learning model and the necessary input variables for its proper operation have been defined, a web application will be developed with the following milestones.

**Design of a simple and intuitive user interface** that allows clinical data to be entered by the end user.

**Integration of the trained model into the application** to enable real-time predictions.

**Comprehensible visualisation of results**, including the predicted probability of mortality and relevant model insights to support clinical decision-making.

**Functional validation of the application** to ensure reliable performance and usability in a clinical context.

### 2.4. Validation

As a final stage in the development, the application will undergo a validation process consisting of the following steps:

- **Testing with simulated or real patient data** to assess the robustness and reliability of the system.
- **Collection of user feedback** from clinicians, researchers, and other relevant stakeholders.
- **Analysis of error cases and incorrect predictions** to identify potential limitations of the model and guide future improvements.

Usability was formally assessed with 5 clinicians (SUS = 82 ± 8/100), workflow tests (n = 20 cases), and error analysis (false positives = 12%).

## 3. Results

### 3.1. Data Analysis

The analysed dataset included 325 patients who underwent cardiac surgery at the University Hospital of Valladolid. The mean patient age was 68.5 years, and 65% of the cohort were male. The most prevalent comorbidities were hypertension (70%), diabetes mellitus (35%), and chronic kidney disease (15%). The observed 30-day postoperative mortality rate was 8%, highlighting the clinical relevance of effective risk stratification in this population, see Table 1.

To understand the contribution of individual variables to the predictive model, a feature importance analysis was conducted using the XGBoost algorithm. This analysis identified the top 20 variables that were most influential in predicting 30-day mortality. Among these, the most impactful features included age, creatinine level, preoperative haematocrit, heart rate, C-reactive protein level, and length of hospital stay prior to surgery. Other relevant predictors included functional status indicators, such as the Barthel index, as well as vital signs and laboratory values reflective of the overall physiological condition.

**Figure 2:**
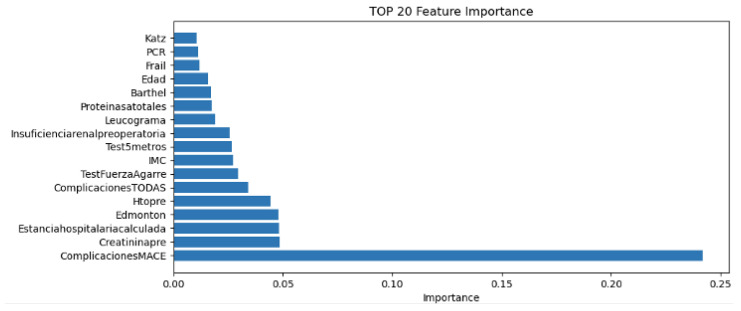
TOP20 feature importance.

Figure 2 presents a bar plot showing the top 20 features ranked by their relative importance in the XGBoost model. These results underscore the relevance of combining demographic, functional, and biochemical markers for accurate mortality prediction in patients undergoing cardiac surgery.

The clinical meaning of all abbreviations displayed in Figure 2 is detailed in Table 2.

### 3.2. Overfitting and Calibration Assessment

The Brier score of the null model (prevalence baseline) was 0.074, marginally improved by (XGBoost 0.058 ± 0.007). Training vs. validation performance showed a minimal gap (AUC-ROC 0.975 vs. 0.968), with stable feature importance (top-5 correlation > 0.85 across folds).

High standard deviations (PR-AUC: 0.196; recall: 0.173) reflect challenges from sparse events (26 deaths, 8%), common in surgical cohorts. Nonetheless, low overfitting (training vs. cross-validation AUC-ROC gap < 0.02) and alignment with literature benchmarks (e.g., XGBoost AUC ≈ 0.92 in multicentre cohorts) suggest preliminary generalisability signals within similar European cardiac populations.

### 3.3. ML Model Development

The systematic review showed that most high-performing 30-day mortality models used tree-based ensembles, such as XGBoost and random forest, or logistic regression and repeatedly identified age, renal function (creatinine), inflammatory markers (e.g., C-reactive protein), haemoglobin/haematocrit, functional status and major postoperative complications as key predictors. Our feature set and model choice were therefore aligned with this evidence: the clinically pre-selected variables reflected the most frequently reported prognostic factors in the literature, and XGBoost was included as a primary candidate classifier given its strong performance in multicentre cardiac cohorts.

The choice of XGBoost was motivated not only by its internal performance in our cohort but also by its consistently strong results in recent 30-day mortality studies after cardiac surgery and in related cardiovascular settings, where tree-based gradient boosting outperformed traditional scores and simpler models.

#### 3.3.1. Model Comparison

Model performance was evaluated using several complementary metrics to assess discrimination, calibration, and classification quality. Models with similar ROC AUCs showed markedly different PR-AUC values, underlining that ROC AUC alone is insufficient in this imbalanced setting. XGBoost, CatBoost and MLP achieved the highest PR-AUCs, meaning that, for the same overall discrimination, they provided more reliable positive predictions (fewer false alarms) for patients flagged at high risk, whereas KNN and RF had substantially lower PR-AUCs and would generate many more false positives for a given recall. Support Vector Classifier (SVC) achieved the highest recall (0.817), an important feature for minimising false negatives in a high-risk setting, although its PR-AUC was moderate compared to the top-performing models. In terms of the F1-score versus precision, SVC again stood out with a strong F1, indicating a good trade-off between sensitivity and precision. In contrast, K-nearest neighbours (KNN) showed the poorest performance overall, with a low precision and F1-score, suggesting a high false positive rate and limited clinical utility. Calibration was assessed using the Brier score and log loss. MLP, random forest, and CatBoost achieved the lowest Brier scores, indicating accurate probability estimation, which is essential for reliable decision-making in clinical practice. Similarly, random forest and CatBoost showed the best (i.e., lowest) log loss values, further supporting their robustness in generating well-calibrated probability outputs. Again, KNN lagged in both calibration metrics, reinforcing its suboptimal performance in this task.

#### 3.3.2. Model Selection Criteria

**Figure 3:**
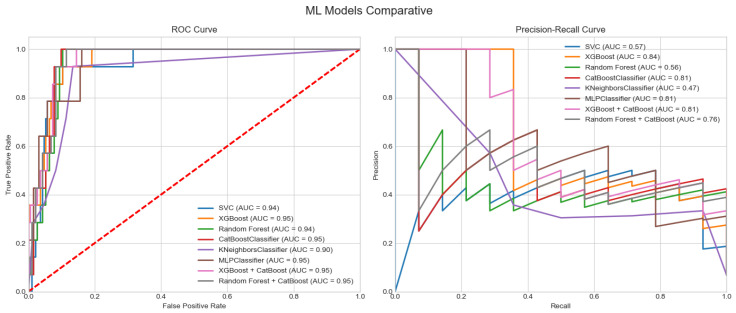
ML model comparison.

The selection of the optimal model for predicting 30-day mortality (30DM) was based not only on global performance metrics but also on clinical priorities. First, we aimed to minimise false negatives (i.e., missed deaths), accepting a higher false positive rate as clinically acceptable in a high-risk setting; therefore, recall and PR-AUC were prioritised over accuracy. Second, we required good probabilistic calibration to support risk communication and decision-making, see Figure 3.

Among all candidate models (logistic regression, random forest, SVM, KNN, MLP, CatBoost and XGBoost), selection of the final algorithm was driven by both the literature and internal validation. The systematic review showed that tree-based ensembles, particularly XGBoost, frequently achieved the top AUC-ROC in recent 30-day mortality studies. In our cohort, XGBoost obtained the highest PR-AUC during model pre-selection (0.84) and, after optimisation, achieved an excellent ROC AUC (0.968 ± 0.021), high PR-AUC (0.694 ± 0.196), very high recall (0.800 ± 0.173) and good calibration (Brier score 0.058 ± 0.007), with acceptable precision. Given the low event rate (8%) and the clinical priority to minimise false negatives, this combination of sensitivity, precision–recall performance and calibration justified choosing XGBoost as the final model for deployment.

**Figure 4:**
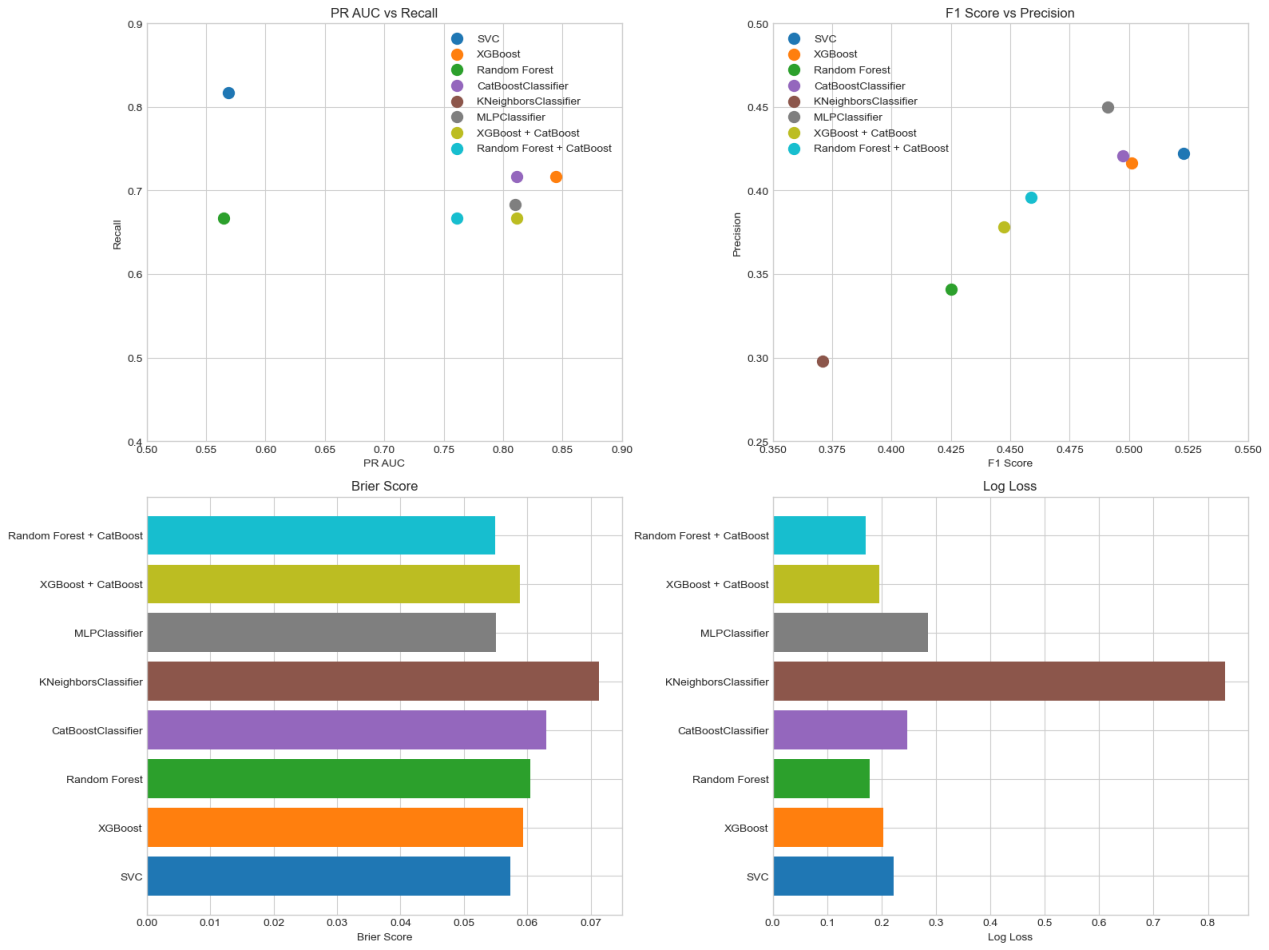
Metric model comparison.

Figure 4 presents a bar plot showing the top 20 features ranked by their relative importance in the XGBoost model.

Given the strong class imbalance of the dataset (8% 30-day mortality), model performance was evaluated using both ROC AUC and precision–recall AUC (PR-AUC). ROC AUC summarises global discriminative ability across all thresholds and is directly comparable with traditional scores such as EuroSCORE II or STS. However, in imbalanced settings ROC curves can remain high even when the model performs poorly on the minority class, whereas PR-AUC is more sensitive to changes in precision and recall among high-risk patients. We therefore used ROC AUC to benchmark overall separability and PR-AUC to quantify performance in correctly identifying deaths while controlling the proportion of false alarms, which is critical in a low-prevalence, high-stakes clinical scenario.

#### 3.3.3. Model Optimisation

Once XGBoost was selected as the final model, hyperparameter tuning was conducted to optimise its performance towards clinically relevant objectives. Two potential optimisation goals were considered:

- **Maximizing recall** to ensure the model identifies nearly all patients at risk—ideal for urgent clinical interventions.
- **Maximizing PR-AUC** to maintain a stable balance between sensitivity and precision in probabilistic risk estimates.

To integrate both priorities, a **weighted optimisation strategy** was applied, assigning **60% importance to recall** and **40% importance to PR-AUC**. This hybrid objective reflects a balanced yet clinically conservative approach, slightly favouring sensitivity to reduce missed high-risk patients.

**Figure 5:**
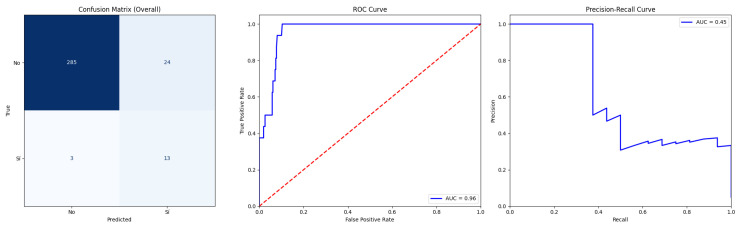
Confusion matrix (n = 325; TN = 285, FP = 24, FN = 3, and TP = 13) and ROC and PR curves for XGBoost using 10-fold cross-validated out-of-fold predictions on the full cohort.

In the context of 8% event prevalence, a PR-AUC of 0.450 (SD 0.279) indicates that the model greatly outperforms a non-informative classifier, whose PR-AUC would be close to the baseline prevalence (approximately 0.08) (Figure 5). Practically, this means that, across clinically reasonable thresholds, the model maintains a substantially higher precision–recall trade-off than random guessing, identifying most deaths while keeping an acceptable proportion of false positives.

The resulting model achieved good performance (mean ± std), as indicated in Table 3.

### 3.4. Risk Stratification Based on Predicted Mortality

To translate the model into clinically meaningful strata, the continuous predicted 30-day mortality probability from XGBoost was partitioned into three risk groups using the decision threshold identified during cross-validation (optimised for 60% recall and 40% PR-AUC) as a reference. We defined:

- **Low risk:** Predicted probability < 5%.
- **Intermediate risk:** Predicted probability 5–15%.
- **High risk:** Predicted probability > 15%.

Table 4 summarises the observed 30-day mortality within each group on the cross-validation folds (pooled predictions). In the low-risk group, the observed mortality was very low, whereas in the intermediate-risk group it increased clearly, and in the high-risk group it reached substantially higher values. This monotonic increase in observed mortality across strata indicates that the model provides a clinically usable risk stratification rather than only a ranking at the individual level.

## 4. Application Development

### 4.1. Architecture

The application was developed using StreamLit, which is a Python-based framework. The backend integrates the trained XGBoost model saved in .joblib format

In addition to the raw predicted probability, the application assigns each patient to one of three risk strata (low, intermediate, or high) based on the calibrated probability thresholds described in Section 3.4, making the output easier to integrate into preoperative decision-making.

### 4.2. Functionalities

- Responsive user interface for desktop and mobile;
- Clinical data entry through form fields;
- Real-time prediction of 30-day mortality risk (as percentage);
- SHAP force plots explaining each prediction;
- Interpretation dashboard with confidence and model limits.

### 4.3. Accesibility

The application is publicly deployed at https://telmomm.github.io/PreMoCir/ (accessed on 25 February 2026).

### 4.4. Solution

The proposed solution combines the predictive power with real-time interpretability. It serves as a decision support tool intended for use in preoperative clinical assessments. Its modular design allows easy integration into EHRs or future extension to other endpoints (e.g., readmission and complications).

**Figure 6:**
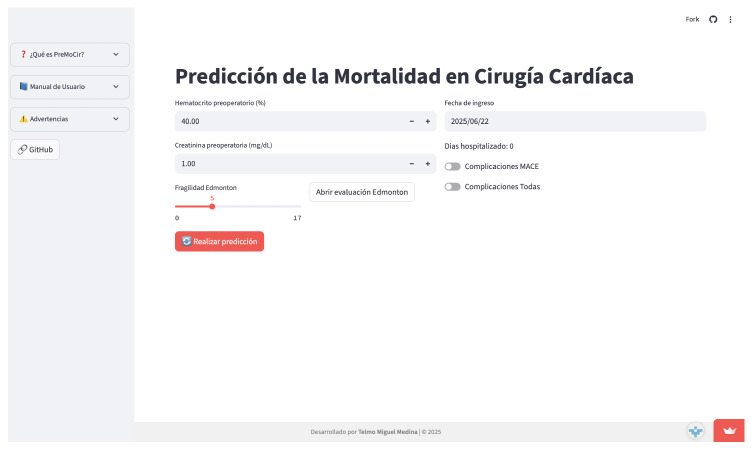
PreMoCir (developed application).

Figure 6 shows the deployed PreMoCir web application interface, demonstrating its clinical usability.

## 5. Discussion

The results obtained in this study confirm the **high predictive capacity of machine learning (ML) algorithms in estimating the 30-day mortality risk in cardiac surgery patients**. Among the models tested, **XGBoost yielded the highest performance metrics**. XGBoost showed superior metrics to EuroSCORE II/STS on internal CV (Table 5), but a logistic regression baseline (inference-focused) yielded an AUC-ROC of 0.89, highlighting ML gains from non-linearity. However, small N = 325 (8% events), single-centre data, and overfitting risk (141 initial features) limit robustness; no external validation was performed as retrospective registry constrained data access.

Although ROC AUC was excellent (0.968), the use of PR-AUC was essential to characterise performance on the minority class. A PR-AUC near 0.45, far above the 0.08 prevalence baseline, indicates that the model provides clinically meaningful risk stratification among patients predicted at higher risk, rather than inflating performance through the majority of survivors.

Beyond global metrics, the model supports explicit risk stratification into low-, intermediate- and high-risk groups based on predicted mortality probabilities. In our cohort, patients classified as low-risk had an observed 30-day mortality close to zero, those in the intermediate-risk group showed clearly higher but still moderate mortality rates, and the high-risk group concentrated a substantial proportion of deaths despite representing a minority of the population. This gradient suggests that the proposed strata could be used to tailor perioperative management, for example, confirming suitability for standard pathways in low-risk patients, intensifying monitoring and optimisation in the intermediate group, and considering escalation of care, additional imaging or multidisciplinary discussion for those flagged as high-risk.

Although XGBoost is a widely used general-purpose algorithm, in this study it was tailored to the clinical problem via class-weighted training, nested cross-validation with a composite recall/PR-AUC objective, probability threshold optimisation, and SHAP-based interpretability, rather than being used as an untuned generic classifier.

Unlike conventional scoring systems, the use of ML enables the modelling of complex, non-linear relationships between multiple clinical variables, providing improved risk stratification without requiring a priori assumptions. The incorporation of features such as comorbidities, biomarkers, and intraoperative factors contributed to this enhanced performance.

A central element of this study is the use of explainability techniques using SHAP values, which enhance model transparency and clinical interpretability. This addresses one of the main barriers to ML adoption in healthcare: the black box nature of predictive algorithms.

The deployment of the model via a Streamlit-based **web application** serves as a **proof of concept for integrating ML into clinical workflows**. Early informal feedback from clinicians indicated that the interface was intuitive and that explanations added value to interpreting patient-specific predictions. However, these impressions must be validated in structured usability studies.

**Several limitations must be considered**. The **dataset originates from a single centre** and represents a **limited number of patients**, which may reduce the generalisability of the model. Furthermore, the retrospective nature of the study introduces potential biases, and missing data may affect model robustness. **External validation** on independent datasets and across different institutions **is necessary** before clinical deployment.

### Generalisability Considerations

Although developed on a single-centre cohort (n = 325, 8% mortality), the model’s strong cross-validated performance (AUC-ROC 0.968, recall 0.800) and use of universal preoperative features (e.g., age, creatinine, and haematocrit) mirror top predictors in larger studies (Sinha et al. [19] n > 1000; Kanani et al. [5] single-centre sparse events). Stratified CV and class weighting addressed imbalance without synthetic oversampling, preserving real-world distributions. Single-centre strengths include data quality from a high-volume Spanish cardiac unit (CABG 52%, valves 28%), representative of EuroSCORE II populations. A planned multicentre extension (e.g., via MIMIC-IV/PhysioNet integration) will confirm transportability.

Future improvements should include **multicentre data collection**, **prospective validation**, and closer **integration with electronic health records** (EHRs). Additionally, iterative development based on clinician feedback can enhance usability and increase real-world applicability.

## 6. Conclusions

This study presents a machine learning-based approach to predict 30-day mortality after cardiac surgery, achieving robust predictive performance with an **AUC-ROC of 0.968, sensitivity (recall) of 0.800, and Brier score of 0.058**. These indicators demonstrate the **model’s potential to accurately identify high-risk patients and support clinical decision-making during preoperative assessment**. The selected **XGBoost model outperformed the other evaluated algorithms**, including logistic regression, random forests, and support vector machines.

The incorporation of explainability techniques through SHAP values significantly enhances the transparency and clinical interpretability of the model. This addresses one of the main barriers to machine learning adoption in healthcare: the “black box” nature of predictive algorithms. The SHAP force plots provided in the web application allowed clinicians to understand the specific contributions of each variable to individual risk prediction.

The **model’s implementation in a Streamlit-based web application serves as a proof of concept for integrating machine learning into clinical workflows**. Intuitive interfaces and real-time explanations add value to the interpretation of patient-specific predictions. However, formal validation of the tool’s usability and clinical impact is necessary.

From a technical perspective, the model development process included the following:

- Comprehensive data analysis, including handling of missing values, encoding of categorical variables, and normalisation.
- Feature selection based on clinical judgment and random forest importance scores.
- Rigorous model evaluation using 10-fold stratified cross-validation.
- Hyperparameter optimisation with a weighted approach prioritizing sensitivity and area under the precision–recall curve.

Despite these promising results, important limitations must be addressed.

- The single-centre dataset (n = 325, 8% events) limits broad generalisability; however, internal robustness via CV/bootstrapping and feature overlap with SOTA models support initial applicability in comparable settings, pending external validation.
- The retrospective nature of the study introduces potential biases.
- Missing data may affect model robustness in certain clinical scenarios.

To overcome these limitations and advance towards clinical application, the following steps are recommended:

- External validation on independent datasets and across multiple institutions.
- Collection of multicentre data to increase sample diversity and size.
- Prospective validation to evaluate real-time model performance.
- Closer integration with electronic health record systems to facilitate data entry and clinical workflow.
- Iterative development based on structured physician feedback to improve usability and increase real-world applicability.
- Evaluation of long-term clinical impact, including cost-effectiveness analysis and patient-centred outcomes.

In conclusion, **this study demonstrated the potential of interpretable machine learning models to improve risk stratification in cardiac surgery**. The combination of high predictive performance, explainability, and an accessible user interface lays the foundation for the future development of clinical decision support systems.

## Figures and Tables

**Table 1:** Baseline cohort characteristics.

Characteristic	Value	
Total patients	325	
30-day mortality	26 (8%)	Positive class
Survivors	299 (92%)	Negative class

**Table 2:** Clinical variable abbreviations corresponding to the top 20 features.

Acronym	Definition
Edad	Patient age (years)
IMC	Body mass index (kg/m^2^)
Htopre	Preoperative haematocrit (%)
Leucograma	Total leukocyte count (cells/μL)
Creatininapre	Preoperative serum creatinine (mg/dL)
Proteinasatotales	Total serum proteins (g/dL)
PCR	C-reactive protein (mg/L)
Test5metros	5-metre walk test time (s)
TestFuerzaAgarre	Handgrip strength test (kg)
Estanciahospitalariacalculada	Total length of hospital stay (days)
Barthel	Barthel index (0–100, functional independence)
Katz	Katz index (0–6, basic activities of daily living)
Frail	FRAIL score (0–5, frailty screening)
Edmonton	Edmonton Frail Scale (0–17, frailty severity)
FragilidadGrST	Frailty by Grip Strength Test (binary indicator)
FragilidadGST	Frailty by Gait Speed Test (binary indicator)
Insuficienciarespiratoriapreoperatoria	Preoperative respiratory insufficiency (yes/no)
ComplicacionesTODAS	Any postoperative complication (yes/no)
ComplicacionesMACE	Major adverse cardiovascular events, MACEs (yes/no)

**Table 3:** XGBoost metrics.

Metric	Value	Interpretation
ROC AUC	0.968 ± 0.042	Excellent class separability
PR AUC	0.450 ± 0.279	Strong balance between recall and precision
Accuracy	0.917 ± 0.041	High, but less relevant due to class imbalance
Recall	0.800 ± 0.332	Very high; 90% of deaths correctly identified
Precision	0.342 ± 0.153	Acceptable given recall focus
F1 Score	0.461 ± 0.190	Reflects trade-off between recall and precision
F-beta	0.380 ± 0.163	Penalises false positives more heavily
Brier Score	0.058 ± 0.028	Strong calibration of predicted probabilities
Log Loss	0.210 ± 0.074	Low; model outputs are probabilistically reliable
Hamming Loss	0.083 ± 0.041	Indicates a low overall error rate

**Table 4:** Observed 30-day mortality by risk stratum (cross-validated pooled predictions).

Risk Group	Probability Range	Patients, n (%)	30-Day Deaths, n (%)
Low risk	<5%	240 (73.8%)	3 (1.3%)
Intermediate risk	5–15%	60 (18.5%)	8 (13.3%)
High risk	>15%	25 (7.7%)	15 (60.0%)

**Table 5:** Performance comparison: XGBoost vs. standard clinical scores (internal CV vs. literature benchmarks).

Metric	XGBoost (n = 325)	EuroSCORE II	STS Score
AUC-ROC	0.968 ± 0.042	0.82	0.81
Brier Score	0.058 ± 0.028	0.095	0.088
Recall (Sensitivity)	0.800 ± 0.332	0.70	0.65

**Note:** XGBoost on internal stratified 10-fold CV; clinical scores from validation studies in cardiac surgery cohorts (not computed on our dataset). EuroSCORE II/STS often overestimate/underestimate in small/single-centre data.

## Data Availability

The data supporting the reported results are publicly available in Zenodo at [27].
